# Oxidized eicosapentaenoic acids more potently reduce LXRα-induced cellular triacylglycerol via suppression of SREBP-1c, PGC-1β and GPA than its intact form

**DOI:** 10.1186/1476-511X-12-73

**Published:** 2013-05-16

**Authors:** Tharnath Nanthirudjanar, Hidehiro Furumoto, Takashi Hirata, Tatsuya Sugawara

**Affiliations:** 1 Division of Applied Biosciences, Graduate School of Agriculture, Kyoto University, Kyoto 606-8502, Japan

**Keywords:** Oxidation, Eicosapentaenoic acid, HepG2, Triacylglycerol, LXRα, SREBP-1c, PGC-1β, GPA

## Abstract

Dietary polyunsaturated fatty acids (PUFA), especially eicosapentaenoic acid (EPA), improve lipid metabolism and contribute to the prevention of vascular diseases such as atherosclerosis. However, EPA in the diet is easily oxidized at room temperature and several types of oxidized EPA (OEPA) derivatives are generated. To compare the efficiencies of OEPAs on lipid metabolism with EPA, human hepatocellular liver carcinoma cell line (HepG2) was treated with EPA or OEPAs and their effects on lipid metabolism related genes were studied. OEPAs more potently suppressed the expression of sterol-responsive element-binding protein (SREBP)-1c, a major transcription factor that activates the expression of lipogenic genes, and its downstream target genes than did EPA under conditions of lipid synthesis enhanced by T0901317, a synthetic liver X receptor (LXR) agonist. Furthermore, PGC-1β, a coactivator of both LXRα and SREBP-1, was markedly down-regulated by OEPAs compared with EPA. The treatment of OEPAs also significantly down-regulated the expression of glycerol-3-phosphate acyltransferase (GPA), the initiating enzyme in triacylglycerol (TG) synthesis, more than EPA. Therefore, the advantageous effects of OEPAs on cardiovascular diseases might be due to their SREBP-1c, PGC-1β and GPA mediated ameliorating effects.

## Introduction

Triacylglycerol (TG), in either the serum or the liver, is a major risk factor for cardiovascular disease (CVD) [1-3]. With respect to the importance of hepatic TG levels, nonalcoholic fatty liver disease (NAFLD) is highly associated with CVD [4-7]. Several prospective epidemiological studies recently demonstrated that both an increased liver enzyme concentration in the serum [8,9] and hepatic steatosis determined by ultrasound [6,7] are able to predict the development of CVD independent of alcohol consumption or traditional CVD risk markers, such as serum LDL cholesterol concentrations. In addition, Rijzewijk et al. demonstrated that type 2 diabetes mellitus patients with high liver TG content showed a decreased myocardial perfusion compared with similar diabetes patients with low liver TG content [3]. Therefore, the dysfunction of hepatic lipid metabolism has been of concern as a therapeutic target of CVD.

Omega-3 polyunsaturated fatty acids (PUFAs), especially eicosapentaenoic acid (EPA), have been developed commercially as dietary supplements due to their various health benefits, particularly their ameliorating effect on CVD [10,11]. EPA has TG-reducing effects in normolipidemic [12] and in hyperlipidemic subjects [13]. It has been proposed that EPA decreases TG through the regulation of peroxisome proliferator-activated receptor α (PPARα) and sterol regulatory element-binding protein (SREBP)-1, which govern hepatic fatty acid (FA) catabolism and synthesis, respectively [14]. It has been well established that SREBP-1c is the major isoform expressed in the liver and in tissues involved in energy homeostasis [15]. Hence, the dysregulation of SREBP-1c has been implicated in the pathogenesis of hepatic steatosis and dyslipidemia, which are closely related to CVD including atherosclerosis [16,17]. SREBP-1c transcription is enhanced by insulin and by agonists of liver X receptor α (LXRα) [18].

Glycerol-3-phosphate acyltransferase (GPA) is supposed to be a rate-limiting step in TG and phospholipid biosynthesis. It catalyzed the first step in glycerophospholipid synthesis by acting as the esterification of glycerol-2-phosphate in the sn-1 position with a fatty acyl-CoA to form 1-acylglycerol-3-phosphate (lysophosphatidic acid). Lysophosphatidic acid is further esterified by 1-acyl-glycerol-3-phosphate acyltransferase (AGPAT) to form 1,2-diacylglycerol-3-phosphate (phosphatidic acid), which is the precursor of TG and phospholipids [19]. The study by Lewin et al. showed that mice deficient in mitochondrial GPA have diminished myocardial TG accumulation during lipogenic diet [20]. Therefore, a decrease of the expression of GPA in the liver might prevent hepatic steatosis and dyslipidemia.

A recent study demonstrated that peroxisome proliferator-activated receptor γ coactivator 1β (PGC-1β) is a potent activator of mitochondrial gene expression, including genes regulating the β-oxidation of fatty acids, but has relatively little ability to stimulate the program of gluconeogenesis [21]. Furthermore, PGC-1β has also been reported to co-activate the LXR and SREBP families and to elevate circulating TG and cholesterol in VLDL particles [22]. As mentioned above, PGC-1β might be a cofactor connecting the regulation of fatty acids with the ameliorating effect on TG accumulation in the liver.

Oxidation products of omega-3 fatty acids, especially EPA and DHA, play crucial roles in the palliative effects via many mechanisms especially those that are cardiovascular-related. A study by Majkova et al. showed that components of oxidized DHA can alleviate the endothelial dysfunction caused by coplanar PCB77 [23]. Furthermore, oxidized EPA (OEPA) has been shown to inhibit leukocyte-endothelial interactions by potently activating PPARα in endothelial cells, and to do so to a much greater extent than native EPA. Although native EPA activates PPARα about half as well as OEPA, unlike EPA, OEPA has effects on leukocyte-endothelial interactions in vitro and in vivo [24]. 5-HEPE, a metabolite produced from EPA in human neutrophils and eosinophils, has been shown to be a potent agonist for G protein-coupled receptor (GPR) 119, which results in a reduction in food intake and in body weight gain in rats [25], and to enhance glucose-dependent insulin secretion [26].

However, the effects of OEPA on many processes are still obscure. Therefore, we aimed to elucidate the mechanism of 4–24 h OEPAs which are composed of various species of oxidation products on lipid metabolism, particularly via the LXRα and SREBP-1c pathway, which plays an important role in lipid metabolism in liver cells. Surprisingly, we found that OEPA significantly down-regulates the expression of lipogenic genes, which results in the suppression of hepatocellular TG more than EPA.

## Results

### Changes in derivatives of EPA oxidation products after auto-oxidation

**Figure 1:**
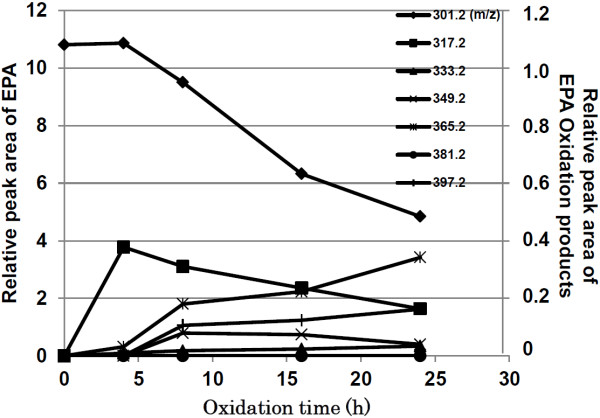
**Ion intensities derived from EPA and EPA oxidation products analyzed by LC/MS.** m/z 301.2 was derived from EPA and m/z 317.2-397.2 were presumably derived from hydroxy- and hydroperoxy-EPAs. Each oxidized EPA was prepared from EPA (10 mg) incubated in 40°C water bath for 4, 8, 16 or 24 h.

First, OEPAs were prepared from EPA (10 mg) by auto-oxidation. [M-H]- at the mass to charge ratio (m/z) 301.2 presented as the molecular ion of EPA and m/z 317.2-397.2 were presumably derived from EPA oxidation products. The intensity of the molecular ion of EPA at m/z 301.2 decreased continuously after auto-oxidation and remained around 50% of the pre-incubation level of EPA at 24 h under these experimental conditions. After oxidation for 4 h, several new ions that were oxidative products of EPA appeared. The molecular ion [M-H]- of hydroxy-EPA (HEPE) at m/z 317.2 reached a peak at 4 h incubation and then gradually decreased. HEPE was the major EPA oxidation product at 4 and 8 h. The ion at m/z 333.2, known as hydroperoxy-EPA, appeared after 4 h of auto-oxidation and increased gradually from 8 to 24 h. On the other hand, the ion at m/z 349.2 was detected at 8 h and after that it reduced gradually. The ions at m/z 365.2 and 397.2 were found at 4 and 8 h, respectively, and then they tended to increase gradually (Figure 1).

### Effects of each fatty acid on HepG2 cell viability

**Figure 2:**
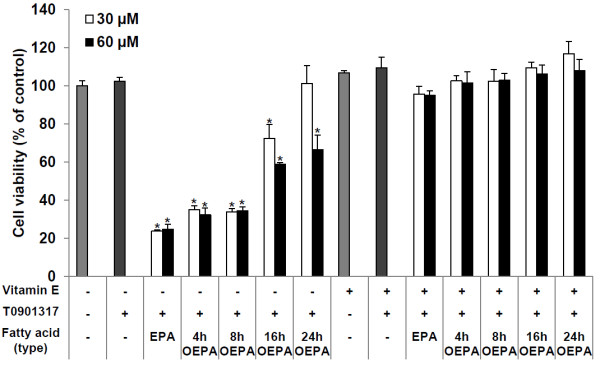
**Effects of EPA and OEPA on cell viability as determined by the water soluble tetrazolium (WST)-1 assay.** Each fatty acid (30 or 60 μM) was added to HepG2 cells with T0901317 (10 nM) and/or vitamin E (10 μM) in serum-free medium containing 0.1% BSA. The final ethanol concentration was 0.3%. After 24 h incubation, the WST-1 solution was added to the cells. The absorbance at 450 nm corresponds to cell viability. Expression levels are presented as relative percentage to vehicle control (ethanol). Data are reported as means ± SD (n = 4/group). * A significant difference from the vehicle control group (p < 0.05).

To ascertain the effects of each compound on cell viability, various concentrations of fatty acids and T0901317 were added to HepG2 liver cells in culture. Figure 2 shows the results of the viability assay after 24 h of treatment. The cytotoxic effect of EPA weakened depending on the oxidation time. EPA, 4 h OEPA, 8 h OEPA and 16 h OEPA at 30 and 60 μM significantly reduced cell viability to around 24%, 34%, 34% and 66% of vehicle-treated control cells, respectively. The 24 h OEPA at 30 μM, but not at 60 μM, had no cytotoxic effect on HepG2 cells under these experimental conditions. Notwithstanding, when HepG2 cells were co-incubated with 10 μM vitamin E, the cytotoxicity of EPA and OEPA was eliminated, which corresponded with the study of Caputo et al. [27].

### OEPA suppresses TG synthesis in HepG2 cells

**Figure 3:**
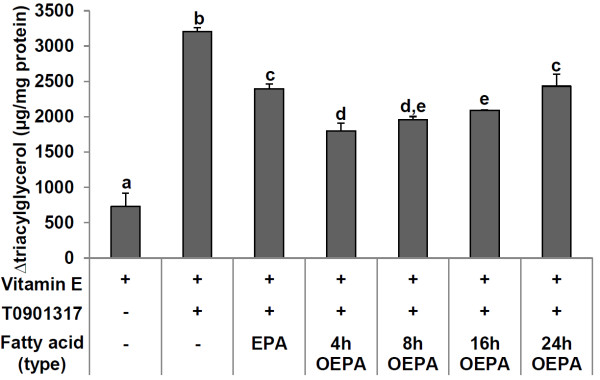
**Effects of EPA and OEPA on TG synthesis in HepG2 cells.** Each fatty acid (60 μM) was added to HepG2 cells with T0901317 (10 nM) and vitamin E (10 μM) as noted in serum-free medium containing 0.1% BSA for 48 h. The final ethanol concentration was 0.3%. The increased TG level per mg protein (TG level after 48 h of incubation minus the level of reference cells) is shown. Data are reported as means ± SD (n = 3-4/group). Values with different letters are significantly different (p < 0.05).

We examined the effects of EPA and each OEPA on TG synthesis in T0901317-induced HepG2 cells. While the cells were being treated with each compound, vitamin E was simultaneously added in FBS-free medium containing 0.1% BSA since EPA has very severe cytotoxic effects, as shown in Figure 2. T0901317 significantly augmented the cellular level of TG (about 3200 μg/mg protein) after 48 h of incubation. Consistent with a previous study [25], EPA significantly reduced TG synthesis of T0901317-treated HepG2 cells to 2400 μg/mg protein. Surprisingly, treatment with OEPA for 4, 8 and 16 h significantly inhibited the cellular TG content of HepG2 cells more than did EPA (Figure 3). Moreover, the 24 h OEPA, which has around 50% the ion intensity at m/z 301.2 to intact EPA (Figure 1), showed almost the same level of TG suppression as did intact EPA (Figure 3). These findings indicate that HEPEs might play a crucial role in the effect of EPA on the inhibition of cellular TG synthesis in HepG2 liver cells.

### OEPA decreases SREBP-1c mRNA expression and maturation more effectively than EPA

**Figure 4:**
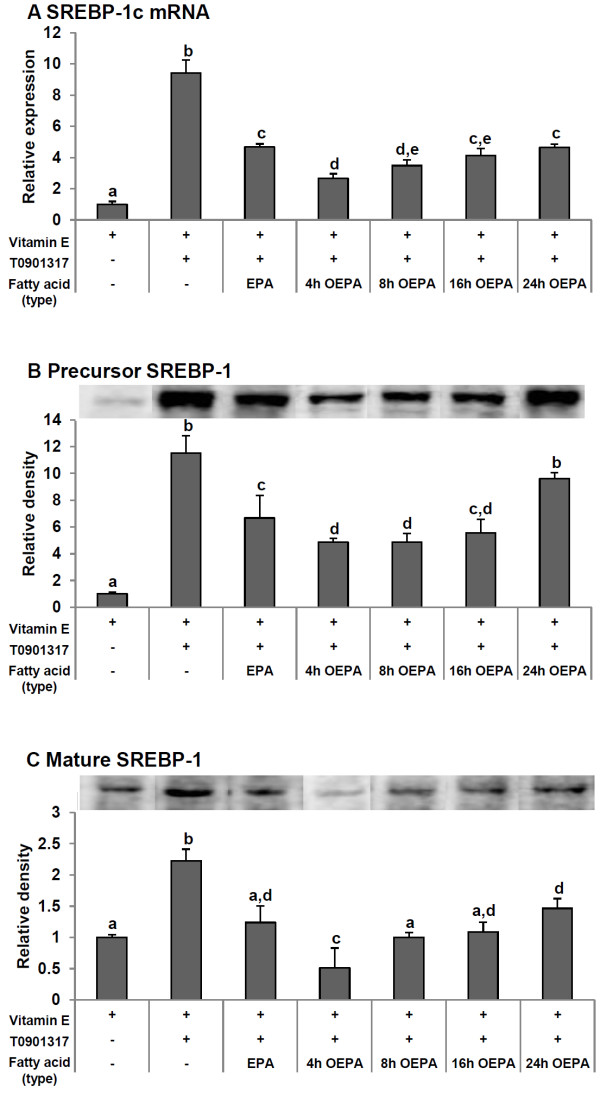
**Effects of EPA and OEPA on the expression of sterol-regulatory element binding protein-1 (SREBP-1). A**: Effect on SREBP-1c mRNA expression. Each fatty acid (60 μM) was added to HepG2 cells with T0901317 (10 nM) and vitamin E (10 μM) as noted in serum-free medium containing 0.1% BSA. The final ethanol concentration was 0.3%. After 24 h incubation, SREBP-1c mRNA levels were quantified by real-time quantitative PCR. Each value of SREBP-1c mRNA was adjusted by that of 18 s rRNA (internal control). Expression levels are presented as -fold induction relative to the vehicle control (vitamin E and ethanol). Data are reported as means ± SD (n = 3/group). Values with different letters are significantly different (p < 0.05). **B,C**: Effects on precursor and mature SREBP-1 protein expression. Each fatty acid was added to HepG2 cells with T0901317 (10 nM) and vitamin E (10 μM) as noted in serum-free medium containing 0.1% BSA. The final ethanol concentration was 0.3%. Representative Western blots are shown. The data represent the mean -fold change of the precursor and the mature forms of SREBP-1 from the vehicle control (ethanol). Data are reported as means ± SD (n = 3-4/group). Values with different letters are significantly different (p < 0.05).

To determine whether the hypolipogenic effect of OEPA on HepG2 cells is due to SREBP-1c, the mRNA expression levels of SREBP-1c and SREBP-1 protein levels were determined. The expression of SREBP-1c mRNA in cells treated with 10 nM T0901317 was 9.4-fold higher than in vehicle-treated control cells (Figure 4A). Treatment with EPA and 4–24 h OEPA significantly down-regulated SREBP-1c mRNA expression stimulated by T0901317. In agreement with the result of TG accumulation, the suppressive effect of OEPA at 4 and 8 h was statistically stronger than EPA. The 4 h OEPA treated cells showed the least SREBP-1c mRNA expression as also noted in the effect on TG synthesis in hepatocytes. Furthermore, the 24 h OEPA reduced SREBP-1c expression to the same level as treatment with EPA (similar to Figure 3).

To investigate the effect of these fatty acids on SREBP-1 protein levels, the full-length precursor form in cell membranes (125 kDa) and the cleaved mature form (68 kDa) in nuclear extracts were estimated by immunoblotting. Because the antibody used cannot distinguish between the SREBP-1c and -1a isoforms, we use the general term SREBP-1 to refer to the results. Representative blots are shown in Figure 4B and C. T0901317 increased the levels of both the precursor and the mature forms of SREBP-1. The 4–16 h OEPA as well as EPA significantly decreased the T0901317 induction of both the precursor and mature forms of SREBP-1. On the other hand, the 24 h OEPA significantly differentiated only mature SREBP-1. Corresponding to SREBP-1c mRNA expression, the precursor form of SREBP-1 was down-regulated by treatment with the 4 and 8 h OEPA more significantly than EPA. Interestingly, the mature form of SREBP-1 was also inhibited by the 4 h OEPA more significantly than EPA and the vehicle control.

### Regulation of mRNA levels of lipogenic genes by OEPA

**Figure 5:**
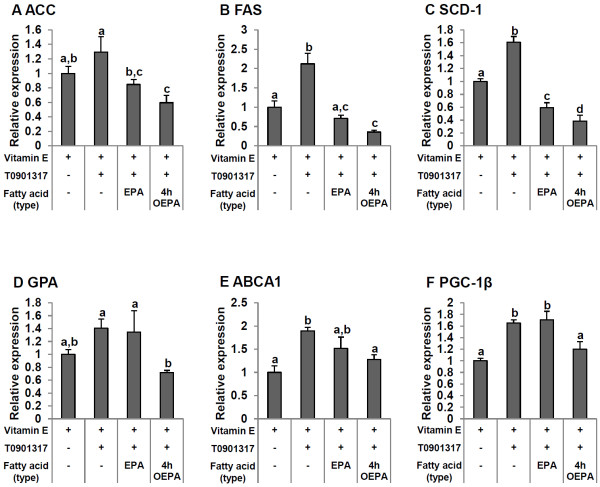
**Effects of EPA and OEPA on the expression of acetyl-CoA carboxylase-1 (ACC; A), fatty acid synthase (FAS; B), stearoyl-coenzyme A desaturase-1 (SCD-1; C), glycerol-3-phosphate acyltransferase (GPA; D), ATP-binding cassette sub-family A member 1 (ABCA1; E) and peroxisome proliferator-activated receptor γ co-activator 1β (PGC-1β; F) mRNAs.** Each fatty acid (60 μM) was added to HepG2 cells with T0901317 (10 nM) and vitamin E (10 μM) as noted in serum-free medium containing 0.1% BSA. The final ethanol concentration was 0.3%. After 24 h incubation, ACC, FAS, SCD-1, GPA, ABCA1 and PGC-1β mRNA levels were quantified by real-time quantitative PCR. Values of ACC, FAS, SCD-1, GPA, ABCA1 and PGC-1β mRNAs was adjusted by that of 18 s rRNA (internal control). Expression levels are presented as -fold induction relative to the vehicle control (vitamin E and ethanol). Data are reported as means ± SD (n = 3/group). Values with different letters are significantly different (p < 0.05).

To further elucidate whether the more efficient effect on the reduction of hepatic TG synthesis by OEPA compared to EPA is due simply to the suppression of SREBP-1c or also to other pathways that might be involved, SREBP-1c target genes and other lipid metabolism related genes were examined. Treatment with EPA or 4 h OEPA significantly decreased the expression of Acetyl CoA carboxylase (ACC), Fatty acid synthase (FAS) and Stearoyl-coenzyme A desaturase-1 (SCD1) mRNAs (Figure 5A,B,C). Corresponding to the aforementioned results, treatment with 4 h OEPA significantly decreased ACC and FAS expression more than the vehicle control while treatment with EPA reduced the expression of these genes to the same level as the vehicle control (Figure 5A,B). Moreover, 4 h OEPA treatment significantly down-regulated the T0901317-induced expression of SCD1 more than did EPA (Figure 5C). GPA, an enzyme located in the endoplasmic reticulum and the mitochondrial membrane that is required for TG synthesis in the glycerol phosphate pathway, which is regulated by SREBP-1c, was significantly weakened by 4 h OEPA compared to LXRα agonist-induced cells, while EPA did not affect the increase of GPA induced by T0901317 (Figure 5D). In agreement with the decreased expression of lipogenic target genes of SREBP-1c, mRNA expression levels of ATP-binding cassette transporter A1 (ABCA1), the other target gene of SREBP-1c that is related to cholesterol transport, was significantly decreased by 4 h OEPA, but was not significantly changed by EPA (Figure 5E). We unexpectedly found that after the 4 h OEPA treatment, the expression of PGC-1β, the co-activator of LXRα and the SREBP families, was significantly down-regulated in the T0901317-induced group. In contrast, EPA did not affect the expression of PGC-1β (Figure 5F).

### Regulation of lipogenic gene mRNA levels by 5-, 11- and 18-HEPEs

From the results mentioned above, the 4 and 8 h OEPAs, which contain high portions of HEPEs (m/z 317.2), showed dominant suppressive effects on the lipogenesis pathway in HepG2 hepatic cells induced by the synthetic LXRα agonist among EPA and other OEPAs which contain lower portions of HEPEs. For this reason, we then used the standards of HEPEs, including 5-, 11- and 18-HEPEs, to identify the kinds of HEPEs that occurred in the auto-oxidized process by LC-MS. The results showed that 5-, 11- and 18-HEPEs might be members of HEPEs in OEPA due to their correlate retention time between peaks of the standards and HEPEs derivative in 4 h OEPA.

**Figure 6:**
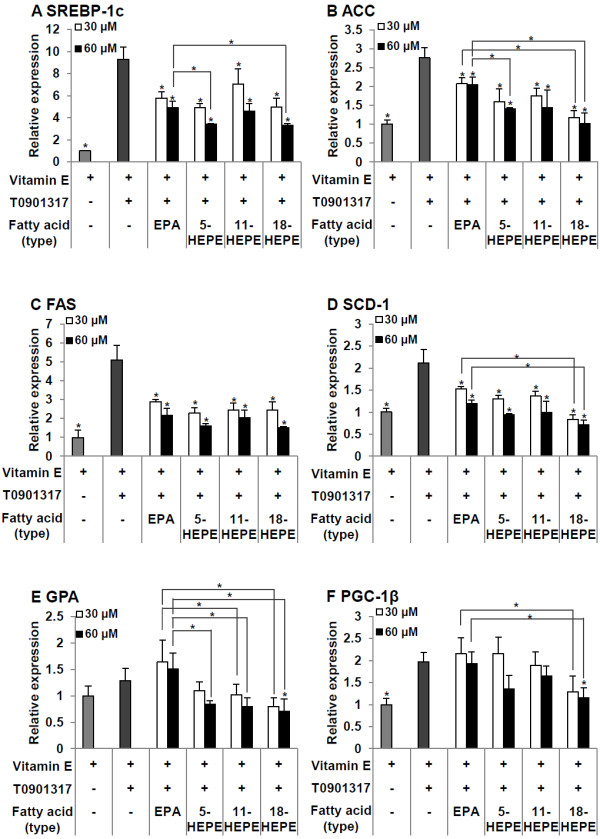
**Effects of EPA, 5-HEPE, 11-HEPE and 18-HEPE on the expression of sterol-regulatory element binding protein-1c (SREBP-1c; A), acetyl-CoA carboxylase-1 (ACC; B), fatty acid synthase (FAS; C), stearoyl-coenzyme A desaturase-1 (SCD-1; D), glycerol-3-phosphate acyltransferase (GPA; E) and peroxisome proliferator-activated receptor γ coactivator 1β (PGC-1β; F) mRNAs.** Each fatty acid (60 μM) was added to HepG2 cells with T0901317 (10 nM) and vitamin E (10 μM) as noted in serum-free medium containing 0.1% BSA. The final ethanol concentration was 0.3%. After 24 h incubation, SREBP-1c, ACC, FAS, SCD-1, GPA and PGC-1β mRNA levels were quantified by real-time quantitative PCR. The value of each mRNA was adjusted by that of 18 s rRNA (internal control). Expression levels are presented as -fold induction relative to the vehicle control (vitamin E and ethanol). Data are reported as means ± SD (n = 3/group). * A significant difference from the T0901317 vehicle control group (p < 0.05).

We then used these standard HEPEs to treat HepG2 cells to confirm whether those HEPEs are involved in the TG reduction of OEPAs. At both 30 and 60 μM concentrations, all fatty acids significantly decreased the augmentation of SREBP-1c expression caused by the LXRα agonist. It was noteworthy that at a 60 μM concentration, 5-HEPE and 18-HEPE significantly reduced SREBP-1c expression more than did EPA (Figure 6A). The expression of SREBP-1c target genes also had a tendency for the same responses. All fatty acids significantly down-regulated the T0901317-induced increase of ACC. At 30 μM, expression of ACC in the 18-HEPE treated group was significantly lower than in the EPA-treated group. In addition, 60 μM 5-HEPE or 18-HEPE significantly reduced the expression of ACC more than did EPA (Figure 6B). Expression of SCD1 at both 30 μM and 60 μM 18-HEPE was significantly lower than was elicited by EPA at similar concentrations (Figure 6D). On the other hand, all fatty acids significantly down-regulated the T0901317-induced expression of FAS, but there was no significant different between any of the fatty acids at the same concentrations (Figure 6C). Among all experimental groups, only treatment with 60 μM 18-HEPE significantly decreased GPA expression compared with T0901317-stimulated cells. Interestingly, cells which were treated with all types of HEPEs in this experiment had significantly reduced expression of GPA more than the EPA-treated cells (Figure 6E). Similar to the 4 h OEPA treatment, 18-HEPE at a high dose can significantly decrease the expression of PGC-1β (Figure 6F).

## Discussion

Previous studies showed that oxidation products of EPA and DHA alleviate some pathways related to CVD [23-26]. In this study, we emphasized the hypolipidemic effect of OEPA on hepatic cells. The hypolipidemic effect of PUFAs, including EPA, is attributable both to a decrease in lipogenesis and an increase in fatty acid catabolism through the regulation of SREBP-1 and PPARα, respectively [14]. Nevertheless, it is well known that PPARα weakly expresses and has very low function in HepG2 cells, because the cells are originated from hepatoma and some kinds of genes, such as PPARα, is mutated [28]. Thus, we focused on the regulation of lipogenesis via SREBP-1 by OEPA in this study.

First of all, we noticed that 4–16 h OEPA significantly decreased the T0901317-induced accumulation of TG in liver cells more than did intact EPA. In this experiment, 4 h OEPA was the most effective agent which was able to suppress TG accumulation. In accordance with that result, the T0901317 induced expression of SREBP-1c, a major transcription factor that activates the expression of lipogenic genes, was significantly down-regulated by 4–8 h OEPA more than EPA. Not only the expression level of SREBP-1c mRNA, but the protein level of SREBP-1c was also significantly inhibited by OEPA more than by EPA. It has been well established that PUFAs suppress SREBP-1c pathway via competing with LXRα ligands in the activation of the ligand binding domain [29-31]. Our previous study showed that trans isomers of EPA (TEPA), similar to EPA, reduces the expression of SREBP-1c through LXRα pathway [29]. Thus, OEPA might decrease the expression of SREBP-1c by competing with LXRα ligands more effectively than EPA.

SREBP-1c target genes were also evaluated after simultaneously treating HepG2 cells with T0901317, and EPA or 4 h OEPA. As expected, ACC, FAS and SCD-1 mRNA expression in the 4 h OEPA-, but not EPA-, treated group was significantly lower than the vehicle control group. Furthermore, 4 h OEPA significantly decreased SCD-1 more than did EPA. Surprisingly, 4 h OEPA significantly diminished the T0901317-induced expression of GPA, but EPA did not. In agreement with our previous finding, the TEPA significantly impaired GPA mRNA expression as well [29]. GPA is the rate limiting enzyme required for the *de novo* synthesis of TG and phospholipids [32,33]. Hence, a decrease of GPA expression in the liver of OEPA might implicate in its preferable hypolipidemic effect. Deletion of the gene encoding SREBP-1c has been shown to result in the failure of T0901317-induced GPA mRNA expression in animal studies [34,35]. However, our previous and present studies illustrate that EPA, which can significantly down-regulate T0901317-induced SREBP-1c expression, cannot abate GPA expression [29]. Therefore, it can be hypothesized that not only SREBP-1c, but also other factors, might regulate GPA expression. Ericsson et al. [36] demonstrated that GPA promoter-luciferase reporter genes are stimulated by the co-expression of SREBP-1a. Importantly, that increase was attenuated when either a dominant negative form of nuclear factor-Y (NF-Y) was cotransfected into the cells or when the GPA promoter contained mutations in the putative binding sites for SREBP-1a or NF-Y. Taken together, modification of the molecular structure of EPA, such as OEPA and TEPA, might be necessary for it to be involved in GPA down-regulation via SREBP-1a or NF-Y.

These results could be also attributable in part to the suppression of PGC-1β expression by 4 h OEPA (Figure 5F), because PGC-1β plays dual roles in modulating hepatic fatty acid metabolism and regulating either fatty acid oxidation or *de novo* fatty acid synthesis by co-activating SREBP-1 and LXRα [22,37]. Our data support the concept that PGC-1β is important for the full induction of lipogenic genes regulated by SREBP-1 and LXRα, and thus, PGC-1β provides a therapeutic target for the metabolic syndrome. It was shown that PGC-1β is a key regulator of hepatic lipogenesis and lipoprotein secretion in response to dietary intake of saturated fats [22]. In contrast, it has minimal effect on the expression of gluconeogenic genes [21]. The expression of PGC-1β in the liver is strongly induced by dietary fats, likely through direct regulation by fatty acids in heptocytes. Hence, the effective inhibition of the cellular TG synthesis by OEPA might be caused by the reduction of PGC-1β expression. Notwithstanding, the mechanism by which OEPA decreases PGC-1β mRNA is unclear, but the reduction of mRNA expression of PGC-1β by OEPA is consistent with the results of our previous report concerning about TEPA [29]. Thus, OEPA and TEPA might suppress the expression of PGC-1β by the same mechanism.

All the aforementioned results confirm that treatment with 4 h OEPA is the most effective in suppressing the hepatic lipogenesis pathway among OEPAs incubated for different oxidation times in this experiment. Interestingly, these inhibition trends are associated with the change in intensity of the ion at m/z 317.2, which appeared as HEPEs in OEPA. Therefore, we assume that HEPEs, but not every oxidation product, might be the most effective oxidation product which plays a crucial role in lipid accumulation in the liver through SREBP-1c pathway inhibition.

It was presumed that HEPEs in the 4 h OEPA includes 5-, 11- and 18-HEPEs by LC/MS analysis compared with HEPEs standards. Similar to the effect of 4 h OEPA, 5- and 18-HEPEs also significantly suppressed the T0901317-induced expression of SREBP-1c, ACC and SCD-1 more than did EPA, while purified 11-HEPE showed almost the same inhibition level as EPA. In this study, 18-HEPE was the most effective in suppressing the expression of SREBP-1c and its target gene mRNAs. Especially, only 18-HEPE significantly down-regulated GPA and PGC-1β compared with T0901317-treated cells.

Of interest, 18-HEPE can be converted from EPA via acetylated cyclooxygenase 2 (COX-2) in vascular endothelial cells after treatment at local sites of inflammation with aspirin, and is then rapidly converted by activated 5-lipoxygenase (5-LOX) in human polymorphonuclear (PMN) leukocytes to insert molecular oxygen and in subsequent steps through 5(6) epoxide formation to bioactive Resolvin E1 (RvE1) and 15-epi-lipoxin (LX) A_5_, the aspirin-triggered lipid mediators hydrolyzed from 5*R*, 6-epoxy-15*R*-HEPE [38,39]. These newly identified chemical mediators appear to exert potent inflammatory and pro-resolving actions both *in vitro* and *in vivo* as proposed by many studies [40,41]. In addition, EPA can be directly metabolized to 5-HEPE in human neutrophils and eosinophils by activated 5-LOX, which has significantly decreased biological effects compared to arachidonic acid-derived metabolites [42,43]. Kogure et al. demonstrated that 5-HEPE is a potent agonist for GPR119 and enhances glucose-dependent insulin secretion [26]. Our study shows for the first time that the location of the hydroxyl group on the carbon backbone of HEPEs is an interesting factor that influences their performance in the regulation of the lipogenesis pathway.

In summary, our data suggest that OEPA augments the ameliorating effect of EPA on lipogenesis in liver cells via the suppression of lipogenic genes related to SREBP-1c. In addition, the preferable ameliorating effect of OEPA on lipogenesis might be due to the decreases of the expressions of PGC-1β and GPA by OEPA, meanwhile EPA does not significantly differentiate those expressions. Our findings provide insight into the importance of the location and the number of hydroxy groups of OEPA in the hypolipidemic effect. This study also suggests that oxidation products as food components might contribute to the beneficial effects of EPA on lipid metabolism in the liver resulting in the prevention of CVD. However, further in vivo experiments are necessary to ensure the effects of dietary OEPA on lipid metabolism.

## Materials and methods

### Materials

Eicosapentaenoic acid and DL-α-tocopherol (vitamin E) were purchased from Nacalai Tesque, Inc. (Kyoto, Japan). T0901317, (±)-5-hydroxy-6E,8Z,11Z,14Z,17Z-eicosapentaenoic acid ((±)5-HEPE), (±)-11-hydroxy-5Z,8Z,12E,14Z,17Z-eicosapentaenoic acid ((±)11-HEPE), and (±)-18-hydroxy-5Z,8Z,11Z,14Z,16E-eicosapentaenoic acid ((±)18-HEPE) were obtained from Cayman Chemicals (Ann Arbor, MI, USA).

### Preparation of OEPA

EPA solution (equivalent of 10 mg) was added into brown glass tubes and the diluted ethanol supernatant was gently evaporated under a N_2_ stream. The prepared tubes were incubated in a 40°C water bath for 4–24 h. After incubation, OEPA was adjusted to a concentration of 100 mM by diluting with ethanol. All samples were stored at −80°C to preserve the quality of the OEPA derivatives.

### OEPA and HEPE analyses by liquid chromatography-mass spectrometry (LC-MS)

For analyzing derivatives of EPA, OEPA and HEPEs, a prominence HPLC system coupled to a LCMS-IT-TOF spectrometer equipped with an electrospray ionization interface (Shimadzu, Kyoto, Japan) was used. A TSK gel ODS-100Z column (2.0 × 50 mm, 3 μm, Tosoh, Tokyo, Japan) was eluted with methanol:water:acetate (70:30:0.01, v/v) at a flow rate 0.2 ml/min. The MS was operated with the following conditions: probe voltage of 1.50 kV, CDL temperature of 200°C, block heater temperature of 200°C, nebulizer gas flow of 1.5 L/min, ion accumulation time of 50 msec, MS range of m/z 250 to 450, and CID parameters were follows: energy, 50%; collision gas 50%.

### Cell culture

HepG2 cells (JCRB 1054; Health Science Research Resources Bank, Osaka, Japan) were cultured in DMEM medium (Invitrogen, Carlsbad, CA, USA) containing 10% fetal bovine serum (Invitrogen, Carlsbad, CA, USA) and antibiotics (100 unit/ml penicillin and 100 μg/ml streptomycin; Gibco Life Technologies Corp, Grand Island, NY, USA) at 37°C in a humidified atmosphere in the presence of 5% CO_2_.

### Lipid extraction and quantification of TG

HepG2 cells were plated on 6-well plates at 5.0 × 10^5^ cells/ml for 24 h in DMEM supplemented with 10% fetal bovine serum and antibiotics as mentioned above. The cells were then treated with EPA, OEPA and/or T0901317 (10 nM) in the presence of vitamin E (10 μM) in serum-supplemented medium. Fatty acids, vitamin E and T0901317 were dissolved in ethanol (final ethanol concentration of 0.3%). After incubation for 48 h, lipids were extracted from cells with chloroform-methanol (2:1, v/v). Reference control cells were extracted for cellular lipids before incubation (zero time control). Collected supernatants were evaporated gently under a N_2_ stream, and TG was quantified using a TG E-test kit (Wako Pure Chemical Industries, Osaka, Japan).

### Determination of mRNA expression levels by real-time RT-PCR

HepG2 cells were seeded in 12-well plates at 2.0 × 10^5^ cells/ml in DMEM supplemented with 10% fetal bovine serum and antibiotics. After 24 h of incubation, each fatty acid was added to HepG2 cells with T0901317 (10 nM) in the presence of vitamin E (10 μM) in serum-free medium containing 0.1% BSA (Sigma-Aldrich, Co., St. Louis, MO, USA). The final ethanol concentration was 0.3%. After 24 h of incubation, total RNA was extracted from the cells using Sepasol reagent (Nacalai Tesque, Kyoto, Japan) according to the manufacturer’s instructions. RNAs were treated with RNase-free DNase (Promega, Madison, WI, USA) to remove contaminating genomic DNA. After inactivating DNase by adding DNase stop solution (Promega, Madison, WI, USA) and heating at 65°C for 10 min, each RNA was transcribed to cDNA using SuperScript RNase II reverse transcriptase (Invitrogen, Carlsbad, CA, USA) with random hexamers at 25°C for 10 min and then at 42°C for 50 min. The reactions were stopped by incubation at 70°C for 15 min. To quantify the mRNA expression level, real-time quantitative RT-PCR was performed in a thermal cycler (Bio-Rad Laboratories, Hercules, CA, USA) using iQ SYBR Green supermix (Bio-Rad Laboratories, Hercules, CA, USA). Primers used for the quantification of each gene are listed in Table 1. Primer pairs were selected to yield gene-specific single amplicons based on analyses by melting curves and by agarose gel electrophoresis. The thermal cycling conditions were as follows: 15 min at 95°C for one cycle, followed by amplification of the cDNA for 43 cycles with melting for 15 s at 95°C and with annealing and extension for 30 s at 60°C. Values were normalized against 18 s rRNA as an endogenous internal standard.

**Table 1:** Real-time RT-PCR primers used for the quantification of human mRNAs

**Gene name**	**Reference or Accession number**	**Forward (from 5’ to 3’)**	**Reverse (from 5’ to 3’)**
SREBP-1c	[45]	GGAGGGGTAGGGCCAACGGCCT	CATGTCTTCGAAAGTGCAATCC
SCD-1	NM_005063	TGGTTTCACTTGGAGCTGTG	GGCCTTGGAGACTTTCTTCC
FAS	[45]	CAGGGACAACCTGGAGTTCT	CTGTGGTCCCACTTGATGAGT
ACC1	NM_198834	ATCCCGTACCTTCTTCTACTG	CCCAAACATAAGCCTTCACTG
ABCA1	[46]	GCAAGGCTACCAGTTACATTTG	GTCAGAAACATCACCTCCTG
GPA	NM_020918	TTGGGTTTGCGGAATGTTAT	GGCAGAACCATCAGGGTTTA
PGC-1β	NM_133263	TGACTCCGAGCTCTTCCAG	CGAAGCTGAGGTGCATGATA
18S	[45]	TAAGTCCCTGCCCTTTGTACACA	GATCCGAGGGCCTCACTAAAC

### Cell viability analysis

Cell viability was assessed by the WST-1 method. HepG2 cells were plated in 96-well culture plates at a density of 1.0 × 10^4^ cells/well in 100 μl DMEM containing 10% fetal bovine serum and antibiotics as detailed above, and were incubated at 37°C for 24 h. Each fatty acid was then added to HepG2 cells with T0901317 (10 nM) and/or vitamin E (10 μM) in serum-free medium containing 0.1% BSA. The final ethanol concentration was 0.3%. After incubation for 24 h at 37°C, 10 μl WST-1 solution (Dojindo Laboratories, Co., Kumamoto, Japan) was added to each well to evaluate cell viability. After incubation for 100 min at 37°C, cell viability was measured using a microplate reader (Molecular Devices Co., Sunnyvale, CA, USA) at a wavelength of 450 nm.

### Cell fractionation and immunoblotting

HepG2 cells were plated in six-well plates at 5.0 × 10^5^ cells/ml for 24 h in DMEM supplemented with 10% fetal bovine serum and antibiotics as detailed above. The cells were then treated with EPA, OEPA and/or T0901317 (10 nM) in the presence of vitamin E (10 μM) in serum-free medium containing 0.1% BSA. After incubation for 24 h, membrane fractions and nuclear extracts from cells were prepared by the method of Hannah et al. [44]. Briefly, cells were harvested by scraping and the cell suspensions were centrifuged at 1,000 × g for 5 min at 4°C. The cell pellets were resuspended in buffer A (250 mM sucrose, 10 mM Hepes-KOH at pH 7.6, 10 mM KCl, 1.5 mM MgCl_2_, 1 mM sodium EDTA, 1 mM sodium EGTA) containing protease inhibitors (Complete Mini Protease Inhibitor tablet, Roche, Mannheim, Germany). The cell suspensions were passed through a 23-gauge needle 20 times and were centrifuged at 1,000 × g for 5 min at 4°C. The pellets were resuspended in 40 ul Buffer B (20 mM Hepes-KOH at pH 7.6, 0.42 M NaCl, 2.5% (v/v) glycerol, 1.5 mM MgCl_2_, 1 mM sodium EDTA, 1 mM sodium EGTA and protease inhibitors). The suspensions were rotated at 4°C for 1 h and were then centrifuged at 10^5^ × g for 15 min at 4°C. The resulting supernatant is designated as the nuclear extract fraction. The supernatant of the original 1,000 × g spin was centrifuged at 10^4^ × g for 15 min at 4°C after which the pellet was dissolved in 25 μl SDS lysis buffer (10 mM Tris–HCl at pH 6.8, 100 mM NaCl, 1% (w/v) SDS, 1 mM sodium EDTA, 1 mM sodium EGTA, and protease inhibitors) and is designated as the membrane fraction. The concentration of soluble proteins in the supernatant was quantified using a DC protein assay kit (Bio-Rad Laboratories, Hercules, CA, USA). For immunoblot analysis, given amounts of membrane fractions (25 μg) and nuclear extracts (10 μg) were separated by 7% and 10% SDS-PAGE, respectively. Protein bands were transferred to polyvinylidene difluoride membranes (Millipore Corporation, Billerica, MA, USA). The filters were probed with a rabbit polyclonal anti-SREBP-1 antibody (H-160, 1:400 dilution; Santa Cruz Biotechnology, Santa Cruz, CA, USA). Bound antibodies were visualized with alkaline phosphatase-conjugated anti-rabbit IgG (1:500 dilution for membrane fractions and 1:1,000 for nuclear extracts; Cell Signaling Technology, Danvers, MA). The bands were visualized with the substrate, chemi-lumione L (Nacalai Tesque, Kyoto, Japan) using a FUJIFILM visualizer (LAS-3000, Fujifilm Corporation, Tokyo, Japan).

### Statistical analysis

Data are reported as means ± SD. Statistical analyses were carried out by one-way ANOVA with Dunnett’s F-test to identify significant difference using Stat View software (SAS Institute, Cary, NC, USA).

## Competing interests

The authors declare that they have no competing interests.

## Authors’ contributions

TN, TH and TS designed the study. TN and HF performed the experiments. TN and TS analyzed the data, wrote the first draft of manuscript and revised the manuscript. All authors read and approved the final manuscript.
